# The evolution of covert, silent infection as a parasite strategy

**DOI:** 10.1098/rspb.2008.1915

**Published:** 2009-03-11

**Authors:** Ian Sorrell, Andrew White, Amy B. Pedersen, Rosemary S. Hails, Mike Boots

**Affiliations:** 1 Department of Animal and Plant Sciences, University of SheffieldWestern Bank, Sheffield S10 2TN, UK; 2 Department of Mathematics, Maxwell Institute for Mathematical Sciences, Heriot-Watt UniversityEdinburgh EH14 4AS, UK; 3 Centre for Ecology and HydrologyMansfield Road, Oxford OX1 3SR, UK

**Keywords:** covert, latent, evolution, disease, model, vertical transmission

## Abstract

Many parasites and pathogens cause silent/covert infections in addition to the more obvious infectious disease-causing pathology. Here, we consider how assumptions concerning superinfection, protection and seasonal host birth and transmission rates affect the evolution of such covert infections as a parasite strategy. Regardless of whether there is vertical infection or effects on sterility, overt infection is always disadvantageous in relatively constant host populations unless it provides protection from superinfection. If covert infections are protective, all individuals will enter the covert stage if there is enough vertical transmission, and revert to overt infections after a ‘latent’ period (susceptible, exposed, infected epidemiology). Seasonal variation in transmission rates selects for non-protective covert infections in relatively long-lived hosts with low birth rates typical of many mammals. Variable host population density caused by seasonal birth rates may also select for covert transmission, but in this case it is most likely in short-lived fecund hosts. The covert infections of some insects may therefore be explained by their outbreak population dynamics. However, our models consistently predict proportions of covert infection, which are lower than some of those observed in nature. Higher proportions of covert infection may occur if there is a direct link between covert infection and overt transmission success, the covert infection is protective or the covert state is the result of suppression by the host. Relatively low proportions of covert transmission may, however, be explained as a parasite strategy when transmission opportunities vary.

## 1. Introduction

Infectious organisms and the diseases that they cause remain a major human health problem, cause significant and sometimes catastrophic damage to agricultural production (Keeling *et al*. 2001) and are increasingly recognized as important determinants of ecological invasions, population dynamics and community structure (Hudson *et al*. 1998; Tompkins & Begon 1999; Tompkins *et al*. 2003). As a consequence, the evolution of their infection strategies and the damage that they cause to their hosts have been the subject of a large body of theory (Anderson & May 1982; Bremermann & Thieme 1989; Bonhoeffer *et al*. 1996; Boots & Sasaki 1999; Brown *et al*. 2002; Dieckmann *et al*. 2002; Day 2003; Boots *et al*. 2004; Gandon 2004). The focus of this theoretical work is to understand what factors determine the various infection strategies that parasites adopt and, in particular, what determines the level of harm that they cause to their hosts. Here, we examine theoretically the factors that may lead to the evolution of covert/silent infections in microparasites.

Infectious organisms show a wide variety of life histories with a wide range of transmission strategies, beyond just horizontal and vertical (parent to offspring) transmissions (Lipsitch *et al*. 1996). In particular, there is an increasing awareness that parasites may produce silent or ‘covert’ infections in addition to the more obvious pathological ‘overt’ infection. Here, when an individual becomes infected, the infection can either become overt causing obvious disease symptoms, and leading to horizontal transmission events, or it may become silent/covert. In the medical community, these infections tend to be referred to as ‘silent’ or ‘dormant’, while in the environmental/virology literature they are referred to as covert. This is an apparently asymptomatic infection that is not horizontally transmitted, where the parasites may, for example, remain within a cell or integrate within the host's chromosomes (Cheung 1991; Delecluse *et al*. 1993; Ahmed *et al*. 1996). In latent virus infections, the viral genome and possibly various virus-encoded products are present, but infectious virus particles are not formed, while a persistent virus infection is characterized by a constant low-level production of virus particles in an infected cell (Dimmock & Primrose 1987). All of these different types of silent/covert infections may or may not have a fitness cost to the host, and in some cases there may be vertical transmission from the infected individual to their offspring. A key characteristic of this strategy is that at some point in the future, these covert infections can become overt, leading to the potential for disease and horizontal transmission. Infections may have a defined ‘latent or exposed’ period, after which they will always convert to an overt infection (susceptible, exposed, infected (SEI) epidemiology), or, by contrast, may convert at some point, but may also never be expressed as overt disease during the lifetime of their host. A distinction should be made between infections that are ‘asymptomatic’ but still horizontally infectious and our definition of covert and silent infections. An asymptomatic infection that causes no or little disease but is still horizontally infectious can be simply understood within the context of it trading off virulence and transmission: if transmission can occur with low or little damage to the host, it will be advantageous. Diseases such as meningitis and many sexually transmitted diseases (STDs) are called silent, but in general the evidence is that they are often still horizontally transmissible. However, it remains a challenge to understand why parasites may evolve to have a non-horizontally transmitted covert stage.

The phenomenon of silent or covert infections is widespread throughout a wide range of different types of infectious organisms. Within human parasites, for example, there is evidence of covert infection in human herpes viruses (Kondo *et al*. 1991) and tuberculosis (TB; Dye *et al*. 1999). Examples of latent viruses include lysogenic bacteriophage infections and the long-term infection of mammalian cells by herpes viruses and varicella zoster virus (Dimmock & Primrose 1987). Most notably, it is estimated that one-third of the human population is infected with *Mycobacterium tuberculosis* (Dye *et al*. 1999); however, only 5–10% of these infections cause overt disease within the first 2 years. Many individuals that have a covert infection will never express the overt, active TB infection that causes disease and has the potential for horizontal transmission (WHO Report 2005). Temperate phages that commonly undergo the lytic cycle (infecting their bacterial hosts horizontally causing host lysis) also rarely lysogenize the host, persisting in a covert state that transmits vertically upon bacterial division (Brown *et al*. 2006). It has also been postulated for a long time that baculovirus infections can persist within their insect hosts without obvious disease symptoms, and recently the use of PCR has demonstrated this phenomenon in a number of experimental systems (Longworth & Cunningham 1968; Jurkovicova 1979; Hughes *et al*. 1993, 1997; Kukan & Myers 1999; Lin *et al*. 1999; Burden *et al*. 2002, 2003, 2006). The development and application of novel detection techniques are likely to find even more examples of these covert infections in wildlife and human pathogens.

Although the implications of covert infection to the population dynamics of their hosts have been examined theoretically (Boots *et al*. 2003; Bonsall *et al*. 2005), the question remains: under what circumstances is covert infection favoured as a parasite strategy? Here, we examine the factors that lead to the evolution of covert infection using evolutionary game theory (adaptive dynamics; Metz *et al*. 1996; Geritz *et al*. 1998) that determines individual parasite strain invasion fitness within explicit ecological dynamics. Our objective is to determine the conditions under which covert behaviour can evolve as a parasite strategy. Our aim is to understand when a silent infection is likely to be a parasite strategy or when it is more likely to be an interaction between the host and parasite. We consider how different assumptions concerning superinfection, and the possibility of protection offered by covert infection, may influence its evolution. We develop a model that includes the possibility of vertical transmission of covert infections and sublethal effects in the fecundity of covertly infected individuals. However, we also relax these assumptions in order to isolate the properties of the evolution of covert infection.

A key question in infectious disease ecology and epidemiology is how do parasites/pathogens persist in the environment when host population sizes, environmental conditions and transmission potential can vary significantly. Persistence may clearly be favoured by vertical transmission, free-living stages that persist in the environment or via a reservoir host, but covert infection has also been hypothesized as a mechanism for persistence in a number of parasites (Burden *et al*. 2003). We therefore use new techniques to examine how variable host population dynamics and seasonality can affect the evolutionary outcome in order to test the hypothesis that covert infection is an evolved parasite strategy in response to a variable environment.

## 2. Models and analysis

We extend the single-strain population dynamic model of covert infection of Boots *et al*. (2003) to consider evolutionary dynamics. The host is divided into a number of classes, *X* is the density of susceptible individuals, *Y*_w_ is the density of individuals infected overtly by the resident strain, *Z*_w_ is the density of individuals infected covertly by the resident strain, *Y*_m_ is the density of individuals infected overtly by the mutant strain and *Z*_m_ is the density of individuals infected covertly by the mutant strain. The interaction between the classes is given by the following system of differential equations:

(2.1a) $$\frac{\text{d}X}{\text{d}t}=aX+a(1-f_{\text{w}})(1-v_{\text{w}})Z_{\text{w}}+a(1-f_{\text{m}})(1-v_{\text{m}})Z_{\text{m}}-\beta_{\text{w}}XY_{\text{w}}-\beta_{\text{m}}XY_{\text{m}}-bX,$$

(2.1b) $$\frac{\text{d}Y_{\text{w}}}{\text{d}t}=(1-p_{\text{w}})\beta_{\text{w}}XY_{\text{w}}-(b+\alpha_{\text{w}})Y_{\text{w}}+c_{\text{w}}Z_{\text{w}}+R_{YW},$$

(2.1c) $$\frac{\text{d}Z_{\text{w}}}{\text{d}t}=p_{\text{w}}\beta_{\text{w}}XY_{\text{w}}+a(1-f_{\text{w}})v_{\text{w}}Z_{\text{w}}-bZ_{\text{w}}-c_{\text{w}}Z_{\text{w}}+R_{ZW},$$

(2.1d) $$\frac{\text{d}Y_{\text{m}}}{\text{d}t}=(1-p_{\text{m}})\beta_{\text{m}}XY_{\text{m}}-(b+\alpha_{\text{m}})Y_{\text{m}}+c_{\text{m}}Z_{\text{m}}+R_{YM},$$

(2.1e) $$\frac{\text{d}Z_{\text{m}}}{\text{d}t}=p_{\text{m}}\beta_{\text{m}}XY_{\text{m}}+a(1-f_{\text{m}})v_{\text{m}}Z_{\text{m}}-bZ_{\text{m}}-c_{\text{m}}Z_{\text{m}}+R_{ZM},$$

where

(2.2a) $$R_{YW}=(1-p_{\text{w}})\beta_{\text{w}}(Z_{\text{w}}+Z_{\text{m}})Y_{\text{w}},$$

(2.2b) $$R_{ZW}=-(1-p_{\text{w}})\beta_{\text{w}}Z_{\text{w}}Y_{\text{w}}-\beta_{\text{m}}Z_{\text{w}}Y_{\text{m}}+p_{\text{w}}\beta_{\text{w}}Z_{\text{m}}Y_{\text{w}},$$

(2.2c) $$R_{YM}=(1-p_{\text{m}})\beta_{\text{m}}(Z_{\text{w}}+Z_{\text{m}})Y_{\text{m}},$$

(2.2d) $$R_{ZM}=-(1-p_{\text{m}})\beta_{\text{m}}Z_{\text{m}}Y_{\text{m}}-\beta_{\text{w}}Z_{\text{m}}Y_{\text{w}}+p_{\text{m}}\beta_{\text{m}}Z_{\text{w}}Y_{\text{m}}.$$

The subscripts ‘w’ and ‘m’ denote the resident and mutant strains, respectively. The host's natural birth and death rates in the absence of disease are denoted *a* and *b*, respectively. The transmission rate of strain *i* is denoted *β_i_*. A proportion (1−*p_i_*) of individuals exposed to the pathogen may develop the disease, in which case they become infective, are subjected to an increased death rate *α_i_* due to the disease, do not recover from the infection and do not reproduce. The remaining fraction *p_i_* of exposed individuals are infected covertly. These individuals are not infectious, do not suffer from an increased death rate and are able to reproduce. Reproduction in these individuals is subject to a reduction in fecundity of *f_i_*. Vertical transmission occurs in a proportion *v_i_* of these births, and these offspring are also covertly infected. The remaining (1−*v_i_*) are born to the susceptible class. Covert infections can become overt at rate *c_i_*. Note there is no sterilization when *f_i_*=0, no vertical transmission when *v_i_*=0 and that when *p_i_*=1 the model behaves as a classic SEI system. The terms *R*_*YW*_−*R*_*ZM*_ in equations (2.2*a*)–(2.2*d*) represent the manner in which superinfection (subsequent infection of an already infected individual) is represented and is described in more detail below. For a wide range of parameters, the population densities of the single-strain model tend to a stable equilibrium with coexistence of the susceptible, overt and covert classes (Boots *et al*. 2003). The evolutionary dynamics for these cases are described in §2*a*,*b*. In §2*c*,*d*, we add seasonality to the model and look at populations that can exhibit cyclic or chaotic dynamics.

### (a) Superinfection

**Figure 1:**
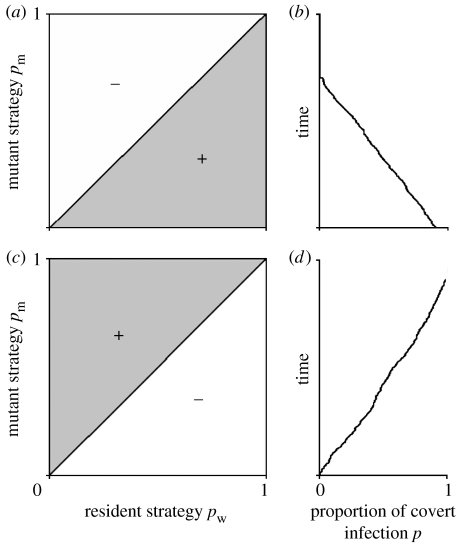
Pairwise invasability plots (PIPs) and simulations of evolutionary dynamics of proportion covertly infected when the underlying population dynamics produce a stable point endemic equilibrium. (*a*,*b*) The evolutionary outcomes where covert infections can be superinfected as defined by the model given by equations (2.1*a*)–(2.1*e*) and (2.2*a*)–(2.2*d*). (*c*,*d*) The outcomes when covert infection is protective and *a*(1−*f*)*v*>*b*. The shaded areas in (*a*) and (*c*) indicate the combinations of *p*_w_ and *p*_m_ for which the mutant will invade from rare. Therefore in (*a*), strains with lower proportions of covert infection can always invade, leading to the evolution of zero covert infection through time as shown in (*b*), while in (*c*) strains with higher proportions of covert infection can always invade, leading to maximization (*d*). For an explanation of how the PIPs were produced, see appendix A; the simulations are explained in the electronic supplementary material.

Previous theoretical studies of superinfection (Nowak & May 1994; Mosquera & Adler 1998; Gandon *et al*. 2001*a*) assume that the outcome of superinfection depends on the difference between the virulence of the strains interacting within the host. In our system, the strains will have the same virulence in the overt class and the covert class is avirulent. Therefore, there are many plausible outcomes of a superinfection event. We tested several alternatives but found the same evolutionary result (see below). First, we analyse the case where superinfection is represented by equations (2.2*a*)–(2.2*d*), these define the outcomes of superinfection of a covertly infected individual when we assume that all reinfections result in the incoming strain taking over and producing either an overtly or covertly infected individual with proportions given by the fraction *p*_w_ or *p*_m_ of the incoming strain. To understand the evolutionary behaviour (the adaptive dynamics), we consider whether a rare mutant strain can invade a resident population at equilibrium. The invasion criteria (see appendix A for details) for a mutant that differs from the resident only in the probability of an infection being covert (a value *p*_m_ for the mutant and *p*_w_ for the resident) is defined as *S*(*p*_m_,*p*_w_) and given by

(2.3) $$S(p_{\text{m}},p_{\text{w}})=cp_{\text{m}}\beta (X^{*}+Z_{\text{w}}^{*})-[(1-p_{\text{m}})\beta (X^{*}+Z_{\text{w}}^{*})-b-\alpha ][a(1-f)v-b-c-\beta Y_{\text{w}}^{*}],$$

where $X^{*},Y_{\text{w}}^{*},Z_{\text{w}}^{*}$ are the resident equilibrium solutions. Invasion occurs if and only if this function is positive. Analysis of equation (2.3) (see equation (A 8)) indicates that mutations are successful if and only if *p*_m_<*p*_w_. Therefore, the only evolutionarily stable strategy (ESS) is that of zero covert infection. Pairwise invasability plots (PIPs) allow this to be visualized clearly (figure 1*a*) since any mutation with a value of *p* less than the current resident strain (values below the diagonal in figure 1*a*) can invade. The result is further confirmed by simulation (figure 1*b*) that indicates that the proportion of covert infection would decrease and disappear over time. Therefore, in the situation where the second infection always takes over, resulting in overt and covert infected hosts of the new strain, covert behaviour will never evolve.

There are many plausible alternatives for representing the outcome of within-host competition between different strains. Another possibility is that all superinfections result in an overt infection of the incoming strain. The system of equations to examine the implications of this phenomenon is again (2.1*a*)–(2.1*e*), but the superinfection terms (2.2*a*)–(2.2*d*) now become

$$R_{YW}=\beta_{\text{w}}(Z_{\text{w}}+Z_{\text{m}})Y_{\text{w}},\;R_{ZW}=-\beta_{\text{w}}Z_{\text{w}}Y_{\text{w}}-\beta_{\text{m}}Z_{\text{w}}Y_{\text{m}},\;R_{YM}=\beta_{\text{m}}(Z_{\text{w}}+Z_{\text{m}})Y_{\text{m}},\;R_{ZM}=-\beta_{\text{m}}Z_{\text{m}}Y_{\text{m}}-\beta_{\text{w}}Z_{\text{m}}Y_{\text{w}}.$$

There is also evidence for other types of covert behaviour. In insect baculovirus systems, it has been shown that asymptomatic, avirulent (covert) infection can be induced into becoming symptomatic and virulent (overt) when the host contracts a rival infection (Burden *et al*. 2003). Alternatively, the results of competition could be decided in other ways. For example, the probability that a parasite strain's infection is covert could determine how often the incoming or the incumbent strain infects the host. A simple way to model this is to have the proportion of infections of the incoming strain becoming covert, determining the winner of the competition. Suitable superinfection terms can be defined for all these cases. We found that the evolutionary dynamics of all these models have the same result as the first model (figure 1*a*,*b*)—namely mutants with a lower proportion of covert infection than the resident will always invade from rare, the only evolutionary stable strategy is zero covert infection, and therefore covert behaviour will not be maintained in the population. Therefore, under a wide range of plausible assumptions about how superinfection occurs, covert infection will never evolve as a parasite strategy.

### (b) Covert infection is protective

There is considerable interest in whether covert infections can protect the host against subsequent infection (Haine 2008). We therefore examine this possibility by assuming that a covertly infected individual is protected, i.e. there is no superinfection, as *R*_*YW*_=*R*_*YM*_=*R*_*ZW*_=*R*_*ZM*_=0. It can be shown (see equation (A 10)) that a mutant differing only in the proportion of covert infection from the resident invades if and only if $(p_{\text{m}}-p_{\text{w}})(a(1-f)v-b)>0$. Therefore, when *a*(1−*f*)*v*<*b*, strains with a lower proportion of covert infection are always favoured and covert infection will not evolve (as in the previous results with superinfection). If *a*(1−*f*)*v*>*b*, such that the rate of vertical transmission is higher than the death rate of the host, evolution favours strains with a higher proportion of covert infection. The ESS is that all infections are covert (figure 1*c*,*d*) and therefore, all overt infections come from conversions of covert infections. As such, in all infections, there is a period of protective covert infection followed by overt infection. This process is equivalent to the SEI epidemiology seen in many disease interactions. When we examine the evolution of the rate at which infections convert from covert to overt (denoted by *c*), we find that the fastest conversion rate will always outcompete the others unless infection is protective and *a*(1−*f*)*v*>*b* (see equation (A 11)). Therefore, SEI-type interactions are never favoured unless the covert infection is protective during the exposed (E) period.

### (c) Host population variation

**Figure 2:**
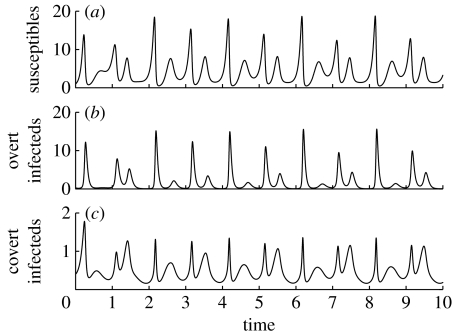
Population dynamics of (*a*) susceptible, (*b*) overt and (*c*) covert individuals with variable host birth rates. The extreme oscillations in the overtly infected population are always present when a non-zero covert infection strategy is favoured. Parameters are *ω*=1, *a*=10, *b*=1, *β*=5, *c*=4, *f*=0.1, *v*=0.9, *δ*=0.8, *α*=25 and *p*=0.09.

**Figure 3:**
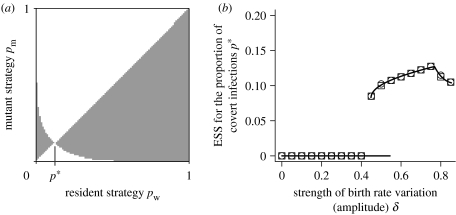
Pairwise invasion and ESS plots for the variable birth rate model. (*a*) The PIP indicates a non-zero ESS proportion of covert infection, p^*^. (*b*) The dependence of this ESS on the strength of variation is shown. The squares and circles are the results of evolutionary simulations starting from an initial strain with a low and high proportion of covert infections, respectively. Note, there are two possible ESSs over a range of values of the amplitude because there are two stable cycles for a monomorphic population at these points. The solution that is attained depends on the initial conditions and therefore which population attractor is approached. (*a*) For the PIP, *δ*=0.7. For both plots, the other parameters are *ω*=1, *a*=10, *b*=1, *β*=5, *c*=4, *f*=0.1, *v*=0.9 and *α*=25. (See the electronic supplementary material for the methods used to determine these results.)

The analysis so far has considered the evolution of covert infection under equilibrium population dynamics. One possibility is that covert infection is a parasite response to variation in host population density. Many organisms show distinct seasonality in their reproduction, leading to population variation. We therefore examine the evolutionary outcome for non-equilibrium population dynamics and include population variability through forcing the birth rate, by substituting *a* with an oscillating term *a*(1+*δ* sin(2*πt*(*ω*)) in equations (2.1*a*)–(2.1*e*). We use equations (2.2*a*)–(2.2*d*) for the superinfection terms in this and the next sections; these terms describe an infection that does not provide protection. The amplitude of the oscillations is controlled by *δ* and the period is *ω*. The population dynamics for this system exhibit oscillatory behaviour, but note that the covert class always has a positive density (figure 2). A PIP (Geritz *et al*. 1998; White *et al*. 2006) of this system for the proportion of covert infection is shown in figure 3*a*. The results indicate that the system with population variability has one ESS that is an evolutionary attractor, p^*^ (and qualitatively similar PIPs occur for all parameter values chosen in this study). We confirm the results by conducting simulations of the evolutionary process (see the electronic supplementary material for details). The position of the ESS, and therefore the proportion of covert infection that will evolve, depends on the forcing amplitude but is always relatively low (ranging between 0 and 0.2; figure 3*b*). When the forcing amplitude is relatively small, covert infection does not evolve, but above a threshold a positive proportion of covert infection becomes an evolutionary stable attractor (figure 3*b*). For some values, multiple evolutionary attractors exist. These occur between the parameter ranges *δ*=0.4 and 0.55 (figure 3*b*), where there are two stable, non-equilibrium, population dynamic attractors. The outcome depends upon initial conditions with either a positive level of covert infection as shown, or zero covert infection (details of how the PIPs are produced and the position of the ESSs is determined are contained in the electronic supplementary material).

**Figure 4:**
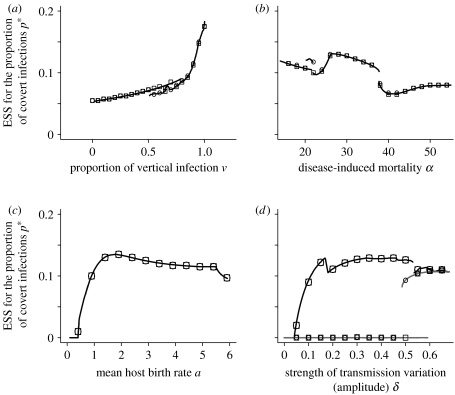
Plots of the ESS of proportion of covert infection against (*a*) proportion of vertical infection, (*b*) disease-induced mortality due to overt infection, (*c*) mean host birth rate and (*d*) strength of transmission variation, where (*a*–*c*) are for variable birth rate and (*d*) is for variable transmission rate. The squares and circles show the result of evolutionary simulations starting from a low and high proportion of covert infections, respectively. The sudden ‘jumps’ in the value of the ESS are caused by transformations in the underlying population dynamics (such as a period doubling bifurcation). For some parameter values, multiple oscillatory population dynamic attractors exist and therefore the value of the ESS attained depends on which population attractor is approached. When not varied as part of the figure, the parameters are *ω*=1, *a*=10, *b*=1, *β*=5, *c*=4, *f*=0.1, *v*=0.9, *α*=25 and *δ*=0.8, except in (*c*) where *b*=0.05 and in (*d*) where for the black line, large squares and circles *a*=1, *b*=0.05 and for the grey line, small squares and circles *a*=10, *b*=1. In (*d*), both sets of parameters result in approximately the same ESS for *δ*>0.6. See the electronic supplementary material for the methods used to determine these results.

The position of the ESS is dependent on the characteristics of both the host and the parasite. Higher proportions of vertical transmission *v* in births from covertly infected hosts lead to higher values of covert infection (figure 4*a*). It is important to note, however; that even with no vertical transmission covert infection can evolve. Vertical infection is beneficial to covert infection as the population declines, but even without vertical infection, covert individuals have lower death rates so still benefit during declines. The presence of vertical infection will make the evolution of covert infection more likely, but is not necessary. The dependency on the level of sterilization acts in the same way as vertical transmission. Covert infection evolves with or without any effects on fecundity, but is more likely when effects are smaller. In figure 4, as in figure 3*b*, there are parameter ranges with multiple, stable, population dynamic attractors and the ESS value depends on which attractor the solutions converge.

The disease-induced death rate affects the ESS (figure 4*b*) in a non-monotonic way. The sudden ‘jumps’ in value of the ESS occur due to changes in the underlying oscillatory population dynamics, which are influenced by the interplay between the mortality parameter, *α*, and the forcing term. The general trend is that increasing disease-induced mortality results in a slight decrease in the evolved value of the proportion of covert infection.

By itself, the longevity 1/*b* of the host has little effect on the position of the ESS. However, the ESS decreases to zero for hosts that produce less than one offspring per complete cycle, i.e. *aω*<1 (figure 4*c*), as we assume positive growth rate these hosts have an average natural lifespan greater than one population cycle, i.e. *ω*<1/*b*. Parasites of long-lived hosts with a low birth rate would not therefore be expected to evolve covert behaviour because of short-term variations in the number of births.

### (d) Variation in transmission

When the birth rate is oscillatory, differences in the parasite transmission rates *β* have no effect on the position of the ESS. However, seasonality may lead to variable transmission rates, due to variation in mixing or mating behaviours for STDs as well as direct environmental effects such as temperature dependence on transmission, and thereby peaks and troughs in the number of infected hosts. We model this using equations (2.1*a*)–(2.1*e*), with a fixed birth rate, and substitute the constant *β* with the oscillating form *β*(1+*δ* sin(2*πt*(*ω*)). The evolutionary outcome, and therefore the PIP produced, is qualitatively similar to the one found for variations in host population size (figure 3*a*) in that for sufficient strength of oscillations covert infection will evolve. When the number of offspring per cycle *aω* is low, a positive proportion of covert infection evolves even for small amplitude variation in transmission (figure 4*d*, black line). If the host has a high birth rate, covert behaviour is not favoured unless the variation in transmission is large (figure 4*d*, grey line).

## 3. Discussion

Understanding the evolutionary determinants of the transmission strategies of parasites and pathogens is crucial to an understanding of how they persist within host populations. A wide range of parasites can produce either overt, infectious disease upon transmission or covert infections, which can be reactivated later in the host's lifetime. We have shown that covert infection is not likely to be an ESS of the parasite in relatively constant equilibrium host populations. There are a number of ways that covert infection can conceivably respond to superinfection, but in all the scenarios examined, covert infection was not favoured. Parasite strains that produce only overt infections always have an advantage in relatively constant host population dynamics. Substantial fluctuations in the host population size or seasonal variation in transmission rates can, however, select for covert infection (even in the absence of vertical transmission from the covert period). Our results therefore suggest that covert strategies may be explained as a parasite response to variation in transmission opportunities, through either seasonal transmission rates or fluctuating host populations. However, our models predict low rates of covert infection, which does not reflect the consistent high levels that are found in some host–parasite systems including TB (Dye *et al*. 1999; WHO Report 2005) and some insect pathogens (Burden *et al*. 2003). These high proportions of covert infection cannot be explained entirely as a parasite strategy to deal with variable opportunities for transmission. We have also shown that if covert infections provide an additional advantage to the parasite, in that they prevent superinfection, they can be favoured even in constant host populations, but only if the vertical transmission rate of the covert strain exceeds the natural death rate of the host. Such protective covert infections will tend to show SEI-type dynamics with all infections being initially covert and reversion after a ‘latent/exposed’ period. Our models are very general in that they include the possibility of vertical infection and different levels of reduced fecundity in the covert class. However, all of our results hold and therefore apply more generally to disease interactions without these processes apart from the crucial role that vertical transmission plays when there is protection.

At first sight, it may appear that a combination of covert and overt infections is a very prudent strategy for the parasite. However, under relatively constant host population dynamics, strains with lower proportions of covert infection always have an advantage. High levels of overt infection are advantageous because a single host has the potential to horizontally transmit to many others, at a rate far exceeding what can be achieved through vertical transmission. In addition, non-protective covert infections are particularly vulnerable to being lost to other strains through multiple infections (van Baalen & Sabelis 1995). Strains with lower proportions of covert infection not only gain more overt infected individuals in the first place, but also gain more through superinfection. We did not consider the situation where superinfection of overtly infected individuals occurs, which could in principle allow covert infection to evolve more easily. However, this assumption is unlikely to affect our results significantly since there will always be a higher risk of superinfection of covert infections due to their longer infectious period. In general, covert infection is at a disadvantage in competition between parasite strains in equilibrium host populations unless it provides protection from superinfection.

Covert infections may be favoured if they prevent superinfection by providing protection to the host, with complete covert behaviour (where all infections are initially covert) favoured if the rate at which covertly infected hosts produce covertly infected offspring is greater than the natural death rate. Therefore strains with protective covert behaviour require high levels of vertical transmission in order to evolve. Whether covert infections are protective in nature is still an open question. Despite the relative paucity of multi-enemy studies, protection and interference have been shown to occur between a number of different parasites. For example, it has been reported that vertically transmitted bacterial symbionts in aphid hosts interfere with the development of the larvae of parasitic wasps (Oliver *et al*. 2003). Viruses have also been shown to affect each others' intracellular replication rate. For example, in *Helicoverpa zea*, it has been found that a slower killing virus (granulosis virus) inhibited the replication rate of a more pathogenic nucleopolyhedrovirus (NPV) in the host, thereby increasing host fitness (Hackett *et al*. 2000). Interestingly when protection leads to covert infection being favoured, there is selection for all new infections to be covert. In effect, this leads to a protective period before overt infection, which may equate to the ‘latent’ period in a disease with an SEI dynamics. In general, such exposed classes may evolve when parasites are protective and transmit vertically during the latent period, but we currently have little information on whether this occurs in nature. At least in some systems, this question could be addressed experimentally, while longitudinal studies within individuals may provide information in other systems.

We have shown that variability in population densities can favour covert behaviour. When the variation is sufficiently large, the host population declines and may sometimes fall below a threshold density at which the overt infection is maintained. Covertly infected individuals survive longer than overtly infected individuals since they do not suffer the effects of disease-induced mortality and may also transmit vertically. The decrease in the density of covert infections is therefore slower than that of overt which tend to fall to lower levels in population declines. Indeed, the covert class tends to follow the dynamics of the susceptible class, which increases after crashes in the overt class. This reservoir of infection allows for a rapid increase in the disease when the host population recovers and therefore strains with covert infection survive these population troughs (figure 2). We consistently found that the ESS was for only a relatively low proportion of covert infection (at most 20% within the wide range of population variations examined here). This relatively low rate occurs due to the balance between the overt infection dominating at high density and selecting for the proportion of covert to move to zero (as in the equilibrium examples) and the covert infection being able to survive and act as a reservoir for infection at low population density. Our models therefore suggest that relatively low rates of covert infection can be explained by host population variation and seasonal forcing. Other mechanisms and processes are required to explain interactions with very high rates of covert infection.

Once there is variation in transmission opportunities, the ecological characteristics of the host–parasite interaction are important in determining the optimal level of covert infection. When the variation in host population is due to changes in birth rate, we find that covert behaviour is unlikely to evolve in long-lived (relative to the cycle length) hosts with low birth rates. We would therefore be less likely to get covert infections in parasites of long-lived slow reproducing organisms such as mammals, particularly if the cycles in their populations are yearly due to seasonal forcing. However, when the variation is due to forcing in transmission rates, we get a striking contrast. Here, covert infection is more advantageous to parasites in hosts with a low birth rate which are relatively long-lived. Indeed in hosts with relatively low birth rates, only relatively small variations in transmission can lead to covert infection being favoured. In high birth rate hosts, parasites would only evolve to become covert if the variation in the transmission rate is large. This emphasizes that it is important to understand the source of the variation to which the host and the parasite is subjected in order to predict the likely level of covert infection. Long-lived, low birth rate organisms obviously including humans, primates and many mammals of agricultural and conservation interest are often exposed to pathogens and parasites that have seasonal transmission rates (Grenfell *et al*. 2002; Koelle *et al*. 2005; Altizer *et al*. 2006; Day & Gandon 2007). Our results suggest that the parasites of this important group of hosts are particularly likely to evolve covert infection as a strategy in response to the variation in transmission rates.

Covert infections have been particularly well studied experimentally in insect viruses. Here, there is good evidence of vertical transmission of the covert virus and of conversion of the pathogen from the covert to pathogenic state (Hughes *et al*. 1993, 1997; Fuxa *et al*. 1999; Burden *et al*. 2003, 2006). The related irido-viruses have very low levels of overt infection (Williams 1998) but high levels of covert infection in natural populations (Williams 1993). In the larval stages of the cabbage moth, *Mamestra brassicae*, PCR-based techniques have detected polyhedrin gene-specific DNA of the *M. brassicae* nucleopolyhedrovirus (MbNPV) from insects derived from long-term laboratory cultures that did not exhibit any overt symptoms of disease. This covert form of the virus was transcriptionally active in the fat body (Hughes *et al*. 1993, 1997) and appears to exist within the host as a persistent, low-level infection. When larvae from this covert-infected stock were fed with heterologous virus, however, the MbNPV was triggered into the overt, lethal disease. This laboratory phenomenon has since been found in field populations (Burden *et al*. 2003). Individuals taken from the field and reared in the laboratory for several generations in virus-free conditions retain the persistent infection, with the occasional individual unpredictably succumbing to the overt disease. Molecular comparisons suggest that the persistent and reactivated overt virus strains are identical (Burden *et al*. 2006). All of these studies provide convincing evidence for high levels of covert infection that is vertically transmitted. Given that these are relatively short-lived, highly fecund hosts, our models suggest that covert infection may be explained by variation in host population density rather than seasonal transmission rates.

In humans, *M. tuberculosis* is particularly well known to persist in a clinically silent state, called latent TB infection (LTBI). Upon host exposure with *M. tuberculosis*, there are three possible outcomes: (i) a small fraction of hosts eliminate the bacterium via an effective innate immune response, (ii) 10 per cent of exposures lead to primary infection and disease manifestation within 2 years of initial exposure (Styblo 1980), and (iii) the vast majority become LTBI cases, or individuals that harbour latent bacilli, which can reactivate after a long, clinically silent hiatus to cause active TB (Bhatt & Slagame 2007). Current estimates suggest that approximately 10 per cent of LTBI cases reactivate to become overt infections (Bhatt & Salgame 2007), and this large proportion of silent/latent hosts create a huge reservoir of future infections; current estimates suggest that roughly one-third of the global population (32%) has a silent or latent form of TB. The silent form could result simply from the immune system suppressing the pathogen in most individuals (Chan & Flynn 2004) or it could be a covert strategy of the parasite as described by our framework. Indeed this possibility has been discussed in the TB literature where the dormant bacteria have been likened to a seed bank for future infection and transmission (Zhang 2004). This latency or dormancy can also occur in other chronic or persistent infections such as streptococcal infections, syphilis and *Heliobacter pylori* (McDermott 1958; Rhen *et al*. 2003). Even if the latency in the parasite is mainly due to the immune system, it is possible that this may still be a parasite strategy through immuno-modulation. For instance, Bhatt & Salgame (2007) suggested that during LTBI, *M. tuberculosis* induces a sufficient Th1 immune response within the host to control its replication but keeps the possibility of reactivation and thus transmission potential under certain altered conditions in the host.

It is important to note that the rates of covert infection in diseases such as TB and in the insect virus examples are much higher than our models would predict in response to variability in host population size or transmission opportunities. Seasonal variation in transmission is likely to occur in human disease and therefore the striking covert strategy of diseases such as TB may indeed be an evolved response, but other factors are needed to explain the high rates of covert infection observed in natural systems. This could include protection from other infections, but there could also be direct links to overt transmission rates through covert infection. Latency and covert infection may result in higher subsequent horizontal transmission rates in some systems, or may in fact be required for any transmission. However, it is also important to recognize that covert infections arise from the interaction between the host and the parasite. There is a clear advantage to the host in controlling the growth rate of the parasite (Miller *et al*. 2005) and thereby reducing the death rate due to infection. Microbial hosts could also use covert infections as anti-competitor strategies. Specifically, Brown *et al*. (2006) investigated bacteria that carry an isogenic temperate phage that acts as a mechanism to invade locally adapted resident populations. The covert phage replicates within the bacterial genome, rarely being induced to release progeny through host lysis. The covertly infected hosts are protected against this horizontal infection, but other susceptible bacteria undergo rapid host lysis when infected, amplifying the transmissible phage particles. Some covert infections may not therefore be parasite strategies, but simply the response of the host to infection. To explore this, host–parasite coevolutionary models must be developed.

In conclusion, we have shown that variability in either transmission rates or host demography and density can select for the evolution of silent/covert infections. There is relatively little theoretical work on the role of variability to the evolution of host–parasite interactions, but adaptive dynamics work in other contexts has suggested that only dramatic variation in dynamics has significant effects on the evolutionary outcomes (White *et al*. 2006). Here we have shown that variability is vital to the evolution of covert infections. Our results apply very generally as they occur with or without vertical transmission and effects on fecundity. There has been considerable interest in the evolution of sterilizing disease, with models predicting that parasites are often selected to castrate their hosts (O'Keefe & Antonovics 2002). It would be an interesting extension to our work to examine the evolution of sterilization in a disease that has covert transmission since this strategy is favoured when there is less sterilization. When there is vertical transmission, there are some interesting parallels with work on the evolution of vertical versus horizontal transmission (Lipsitch *et al*. 1996). However, vertical transmission is only important to the evolutionary process when covert infections are protective, and our main conclusion that variability is needed for the evolution of covert infection in the absence of protection does not depend on the presence of vertical transmission.

## Appendix A

### A.1 Equilibrium population dynamics

Here we explain the invasion criterion used to determine the evolutionary results and to produce the PIPs when the underlying population dynamics exhibit a stable point equilibrium (figure 1), and the methods used for non-equilibrium dynamics are detailed in the electronic supplementary material. We use Jacobian analysis, taking the order of the variables to be (*X*,*Y*_w_,*Z*_w_,*Y*_m_,*Z*_m_). Determining invasion by a rare mutant is equivalent to analysing the stability of the equilibrium $\left(X^{*},Y_{\text{w}}^{*},Z_{\text{w}}^{*},0,0\right)$. The Jacobian matrix evaluated at this point has the form

$$J=\left(\begin{array}{cc} A & B\\ O & C \end{array}\right),$$

where *A*, *B* and *C* are submatrices of size 3×3, 3×2 and 2×2, respectively, and *O* is the 2×3 zero matrix. The matrices *A* and *C* are linearly independent. This means that the stability conditions can be determined from examining *A* and *C* separately. The matrix *A* corresponds to the stability of the resident strain in the absence of the mutant strain. The parameters of the resident are chosen such that the equilibrium of susceptibles and resident strain considered in the absence of the mutant is always stable (Boots *et al*. 2003). The matrix *C* corresponds to the stability with respect to invasion of the rare mutant. Thus, to determine invasion we need to consider only the stability of *C*.

Here, we shall consider the model represented by equations (2.1*a*)–(2.1*e*) and (2.2*a*)–(2.2*d*), but the process is similar for the other combinations of superinfection. The matrix *C* is given by

(A1) $$C=\left(\begin{array}{cc} (1-p_{\text{m}})\beta_{\text{m}}(X^{*}+Z_{\text{w}}^{*})-b-\alpha_{\text{m}} & c_{\text{m}}\\ p_{\text{m}}\beta_{\text{m}}(X^{*}+Z_{\text{w}}^{*}) & a(1-f_{\text{m}})v_{\text{m}}-b-c_{\text{m}}-\beta_{\text{w}}Y_{\text{w}}^{*} \end{array}\right).$$

Its stability can be determined using the Routh–Hurwitz conditions. For the equilibrium point to be stable, the trace(*C*)=*τ*(*C*)<0 and the determinant(*C*)=*Δ*(*C*)>0. If one or both of these inequalities are violated, then the equilibrium is unstable and the mutant can invade from rare.

For the matrix *C* above, the conditions for stability are

(A2) $$\tau (C)=(1-p_{\text{m}})\beta_{\text{m}}(X^{*}+Z_{\text{w}}^{*})-b-\alpha_{\text{m}}+a(1-f_{\text{m}})v_{\text{m}}-b-c_{\text{m}}-\beta_{\text{w}}Y_{\text{w}}^{*}<0,$$

(A3) $$\Delta (C)=[(1-p_{\text{m}})\beta_{\text{m}}(X^{*}+Z_{\text{w}}^{*})-b-\alpha_{\text{m}}][a(1-f_{\text{m}})v_{\text{m}}-b-c_{\text{m}}-\beta_{\text{w}}Y_{\text{w}}^{*}]-c_{\text{m}}p_{\text{m}}\beta_{\text{m}}(X^{*}+Z_{\text{w}}^{*})>0.$$

Starting from a stable point of parameter space, the condition on the determinant (A 3) will be the first to fail. Therefore, the invasion criteria can be determined from the determinant condition alone. This is true for all the other models considered here. From (A 3), we define the invasion criteria for a mutant that differs from the resident only in the parameter *p* by

(A4) $$S(p_{\text{m}},p_{\text{w}})=cp_{\text{m}}\beta (X^{*}+Z_{\text{w}}^{*})-[(1-p_{\text{m}})\beta (X^{*}+Z_{\text{w}}^{*})-b-\alpha ][a(1-f)v-b-c-\beta Y_{\text{w}}^{*}],$$

where the subscripts have been dropped from the other parameters. If *S*(*p*_m_,*p*_w_)>0, then the mutant can invade the resident equilibrium. However, we also know that

(A5) $$S(p_{\text{w}},p_{\text{w}})=cp_{\text{w}}\beta (X^{*}+Z_{\text{w}}^{*})-[(1-p_{\text{w}})\beta (X^{*}+Z_{\text{w}}^{*})-b-\alpha ][a(1-f)v-b-c-\beta Y_{\text{w}}^{*}]=0,$$

since the introduction of the ‘w’-type neither grows nor decreases at its own attractor. Thus,

$$S(p_{\text{m}},p_{\text{w}})=S(p_{\text{m}},p_{\text{w}})-0=S(p_{\text{m}},p_{\text{w}})-S(p_{\text{w}},p_{\text{w}}).$$

Subtracting (A 5) from (A 4) and factorizing allows one to show that

(A6) $$S(p_{\text{m}},p_{\text{w}})=(p_{\text{w}}-p_{\text{m}})(\beta Y_{\text{w}}^{*}-a(1-f)v+b)\beta (X^{*}+Y_{\text{w}}^{*}).$$

Solving the system of equations (2.1*a*)–(2.1*e*) for steady states, the equilibrium point satisfies

(A7) $$X^{*}=\frac{a(1-f)(1-v)Z_{\text{w}}^{*}}{\beta Y_{\text{w}}^{*}-a+b}.$$

The positivity of *X*^*^ implies that the denominator of (A 7) is positive, with (A 6) this gives

(A8) $$S(p_{\text{m}},p_{\text{w}})=K(p_{\text{w}})(p_{\text{w}}-p_{\text{m}}),$$

where *K*(*p*_w_) is a positive function when a stable equilibrium exists. Therefore, the mutant invades if and only if *p*_m_<*p*_w_. Figure 1*a* indicates regions where equation (A 8) is positive and the mutant can invade, and where it is negative and mutant invasion is unsuccessful.

When the covert infection offers protection, one can redo the above calculation with *R*_*YW*_=*R*_*YM*_=*R*_*ZW*_=*R*_*ZM*_=*0* in equations (2.1*a*)–(2.1*e*) and find an explicit formula for *X*^*^ in terms of the parameters alone. The invasion function becomes

(A9) $$S(p_{\text{m}},p_{\text{w}})=\frac{\left(p_{\text{w}}-p_{\text{m}})(b+\alpha )(b-a(1-f)v)(b+c-a(1-f)v\right)}{(1-p_{\text{w}})(b-a(1-f)v)+c}.$$

At equilibrium, (2.1*c*) implies that (*b*+*c*−*a*(1−*f*)*v*) is positive. Therefore,

(A10) $$S(p_{\text{m}},p_{\text{w}})>0\Leftrightarrow (p_{\text{w}}-p_{\text{m}})(b-a(1-f)v)>0$$

and so when *a*(1−*f*)*v*>*b*, then equation (A 9) is positive if *p*_m_>*p*_w_ and covert infection is favoured. When *a*(1−*f*)*v*<*b*, the opposite is true and covert behaviour does not evolve.

The same process can be carried out for mutants that differ only in the rate of conversion from covert to overt, i.e. *c*_m_≠*c*_w_. The invasion criterion in the case that covert is protective is given by

(A11) $$S(c_{\text{m}},c_{\text{w}})=\frac{\left(c_{\text{m}}-c_{\text{w}})(b+\alpha )p(b-a(1-f)v\right)}{(1-p)(b-a(1-f)v)+c_{\text{w}}}.$$

This shows that mutants with a slower conversion rate, *c*_m_<*c*_w_, can invade if and only if *a*(1−*f*)*v*>*b* using the same argument as above. The invasion criterion when superinfection is represented by (2.2*a*)–(2.2*d*) is $S(c_{\text{m}},c_{\text{w}})=(c_{\text{m}}-c_{\text{w}})(\beta (X^{*}+Z_{\text{w}}^{*})-(b+\alpha ))$. Using (2.1*a*)–(2.1*c*), it is possible to show that $(\beta (X^{*}+Z_{\text{w}}^{*})-(b+\alpha ))>0$. Therefore, only mutants with a faster conversion rate than the resident can invade.

## Supplementary Material

Non-equilibrium dynamics and numerical simulations Here we provide details of how the results were determined for the non-equilibrium cases and give details of the numerical simulations.
