# Supplementary material for: The evolution of covert, silent infection as a parasite strategy
Source: Proc Biol Sci. 2009 Mar 11;276(1665):2217–26. doi: 10.1098/rspb.2008.1915 (PMC2677597; doi:10.1098/rspb.2008.1915)
Supplement: Non-equilibrium dynamics and numerical simulations — Here we provide details of how the results were determined for the non-equilibrium cases and give details of the numerical simulations. [file rspb20081915s14.doc]

## Electronic supplementary material to

**The Evolution of Covert, Silent Infection as a Parasite Strategy**

Ian Sorrell^a^, Andrew White^b^, Amy B. Pedersen^a^, Rosemary S. Hails^c^ and Mike Boots^a^

## Here we provide details of the methods used to find the results for the non-equilibrium population dynamics cases and give details of the numerical simulations.

## Non-equilibrium population dynamics

It is rarely possible to determine an algebraic expression for the invasion exponent when the underlying population dynamics are non-equilibrium (periodic, quasi-periodic or chaotic attractors). Instead computational techniques can be used to determine the invasion exponent (Metz *et al.* 1992, White *et al.* 2006). The pairwise invasion plot figure 3a was created by numerically determining the invasion exponent as the largest Lyapunov exponent using the following procedure (Christiansen & Rugh 1997). The first three equations of the system shown below are from equations (1) and (2) with zero mutant type. The other equations determine the stability of the system to invasion from a rare mutant.

where

is the matrix (A1) (in the appendix of the main document) with time dependent variables, is the Euclidean product, , *e*_1_*, e*_2_ are time dependent, two dimensional vectors with orthonormal initial conditions, for example *e*_1_(0) = (1,0), *e*_2_(0) = (0,1), and *l*_1_, *l*_2_ , are real numbers with *l*_1_(0) = *l*_2_(0) = 0. These equations are solved for a time period of length *T* with initial condition for (*X*, *Y_w_*, *Z_w_*) on the resident population attractor. The Lyapunov exponents for the mutant are then calculated as

with the largest of these the invasion exponent. The exact calculation of *λ_j_* occurs in the limit as $T\to\infty$ (Christiansen & Rugh 1997) but a good approximation can be achieved for large *T*. Figure 3 a shows a PIP indicating where the invasion exponent is positive or negative which is equivalent to whether mutant invasion is successful or unsuccessful respectively. Such PIPs can be used to understand the evolutionary behaviour and determine the value of the ESS as other parameters vary (figures 3 b and 4). We implemented the scheme using the matlab solver ode45.

In the majority of cases the resident population attractor is periodic. In these cases, to ensure that the value of the ESS determined using the above method is correct, we additionally employ a method that calculates the Floquet multipliers of the periodic system using the AUTO-07P continuation and bifurcation software (Doedel *et al.* 2006). The software can be used to find the stable resident population attractors and determine their period *P.* It can then determine the stability of the resident attractor to invasion by a rare mutant by calculating the Floquet multipliers *ρ_i_*. The Lyapunov exponents are given by

where the largest of these is the invasion exponent. In all cases the Floquet and large *T* methods were found to be in correspondence. Note that to use AUTO the system of equations (1) and (2) with must be made autonomous. This can be done by use of

with *R*(0) = 1, *S*(0) = 0, this gives *S*(*t*) = sin(2*πt / ω*).

*Simulation methods*

Further verification of the value of the ESS was achieved by simulating the evolutionary dynamics of the model system. The figures 1 b and 1 d and the squares and circles in figures 3 and 4 were produced by numerically solving the population dynamics for a fixed time (t) starting from a monomorphic population. A mutant strain was generated at low population density with a parameter value close to that of a current strain in the population (which was selected based on the relative density of the current strains). The population dynamics are then solved for a further time t. Any strain whose population drops below a (low) threshold are excluded and then a new mutant is generated from the remaining strains. This therefore simulates the evolutionary process and has been shown to correctly approximate the evolutionary behaviour predicted by adaptive dynamics (White *et al.* 2006).

REFERENCES

Christiansen, F. & Rugh, H.H. 1997 Computing Lyapunov spectra with continuous Gram–Schmidt orthonormalization, *Nonlinearity* **10**, 1063–1072.

Doedel, E.J. et al. computer software AUTO-07P, <http://indy.cs.concordia.ca/auto>

Metz, J.A.J., Nisbet, R.M. and Geritz, S.A.H. 1992. How should we define ‘fitness’ for general ecological scenarios? *Trends in Ecology and Evolution* **7**, 198-202.

White, A., Greenman, J.V., Benton, T.G. & Boots, M. 2006. Evolutionary behaviour in ecological systems with trade-offs and non-equilibrium population dynamics. *Evolutionary Ecology Research*, **8**, 387-398.
